# From Eyesight to Insight: Addressing Barriers and Facilitators of Physical Activity Among Indian Elders

**DOI:** 10.1016/j.dialog.2026.100290

**Published:** 2026-03-06

**Authors:** Nawaj Mehtab Pathan, Vibhuti Tiwari, Rucha Agnihotri, Dr. V. Sarath Babu, Dr. Prerana Dalvi

**Affiliations:** a Department of Neurophysiotherapy, MGM Institute of Physiotherapy, Chh. Sambhajinagar-Affiliated to MUHS Nashik, India; b Department of Cardiovascular Respiratory Physiotherapy, MGM Institute of Physiotherapy, Chh. Sambhajinagar-Affiliated to MUHS Nashik, India; c Department of Neurophysiotherapy, Dhaneshwari College of Physiotherapy, Chh. Sambhajinagar, India; d MGM Institute of Physiotherapy, Chh. Sambhajinagar-Affiliated to MUHS Nashik, India

**Keywords:** Physical activity, Elders, Barriers, Facilitators, Lifestyles

## Abstract

Physical inactivity among Indian elders represents a significant public health challenge, influenced by diverse barriers such as fear of falls, limited social support, and environmental constraints. This viewpoint explores the interplay of these barriers and facilitators, advocating for a paradigm shift from focusing solely on functional limitations (“eyesight”) to addressing psychological, social, and environmental determinants of physical activity (“insight”). By integrating insights from geriatric health, urban planning, and behavioral sciences, this article underscores the importance of culturally tailored interventions. Recommendations include community-driven programs, inclusive urban designs, and caregiver engagement to foster sustained participation in physical activities. A holistic approach is crucial to empower Indian elders, improve their quality of life, and mitigate the burden of non-communicable diseases in aging populations.

## 1 Introduction

Physical activity plays a crucial role in maintaining health and well-being for the elderly population. Regular physical activity has significant benefits on both physical and cognitive functioning among older adults. Regular physical activity improves cardiovascular and musculoskeletal health, preserves functional independence, and supports cognitive and psychological well-being, making it a cornerstone of healthy aging [1], [2], [3], and Promoting physical activity among older adults should therefore be a priority in health promotion efforts [4].Engaging in regular physical activity is crucial for healthy aging. The benefits of physical activity are wide-ranging, from improving cardiometabolic health and physical fitness to enhancing mental health outcomes. Therefore, promoting physical activity among older adults should be a priority in health promotion efforts [5].

Aging is associated with changes in multiple physiological systems, including mitochondrial dysfunction, cellular senescence, autophagy, epigenetic alterations, and microbiome disturbances [6].These changes can lead to reduced functional capacity, increased susceptibility to chronic diseases, and the development of geriatric syndromes such as frailty, falls, and cognitive decline [7].

Exercise therapy is often recommended as an effective intervention for maintaining mental health and combating challenges associated with sedentary lifestyles [8]. This study is conceptually grounded in the Social Ecological Model, which emphasizes how individual, interpersonal, community, and policy-level factors interact to shape physical activity behaviors. Applying this model allows a clearer understanding of how psychological, social, and environmental determinants collectively influence the activity levels of Indian elders.

However, the overall evidence supports that the risk-to-benefit ratio generally favors continuing regular aerobic exercise [9]. Regular physical activity and exercise are powerful lifestyle strategies that can mitigate the risk of developing various chronic conditions, including breast cancer and its recurrence [10]. It improves cardiovascular health, preserves muscle mass and strength, enhances bone density, supports brain health, and alleviates symptoms of depression and anxiety. The benefits extend to older adults, improving their quality of life by enhancing cardiovascular and musculoskeletal health, cognitive function, and mental well-being [11].

Hence, regular physical activity is essential for enhancing and maintaining both physical and mental health across the lifespan [12]. Engaging in physical activity plays a crucial role in promoting healthy aging by supporting cognitive health, alleviating depression, and fostering social interactions among older adults. Consistent participation in exercise has been linked to improvements in memory, attention, and executive functioning, which may help prevent or delay cognitive decline [13]. Furthermore, staying physically active contributes to better mental well-being by alleviating symptoms of depression and enhancing emotional resilience [14].

Engagement in physical activity programs can foster social connections and build community relationships, addressing the risk of social isolation often faced by older adults [15]. Interestingly, stereotypes about physical aging can influence older adults' engagement in moderate-intensity activity [16]. This suggests that addressing negative stereotypes could potentially increase participation in physical activity among older adults. Additionally, the built environment plays a crucial role in supporting or impeding older adults' physical and social engagement, highlighting the importance of designing supportive environments to facilitate activity [17].

Despite growing recognition of the importance of physical activity for healthy aging, a significant gap persists in the literature and in practice. Much of the existing focus—both in research and interventions—remains on overcoming functional limitations (the “eyesight” of aging), such as poor mobility or chronic disease. However, for Indian elders, participation is equally, if not more, constrained by psychological, social, cultural, and environmental determinants (the “insight” into aging). There is a pressing need for a conceptual shift that moves beyond a biomedical model to embrace an ecological, culturally grounded perspective. This viewpoint article addresses this gap by synthesizing evidence across disciplines to advocate for a holistic framework tailored to the unique socio-cultural context of aging in India.

## 2 Purpose & Conceptual Approach

The purpose of this article is to eloquent a multi-level, culturally sensitive framework for endorsing physical activity among Indian elders by recognizing key barriers and facilitators. This is a conceptual synthesis grounded in the Social Ecological Model (SEM), which posits that health behaviors are inclined by a dynamic interplay of individual, interpersonal, community, and societal factors. This contains a narrative synthesis of contemporary literature from geriatric health, behavioral science, urban planning, and public policy. We aimed specifically on studies and data relevant to the Indian context to propose actionable, integrated recommendations that align with global health agendas.

## 3 Barriers To Physical Activity

Despite the numerous benefits, older adults face several barriers to engaging in physical activity. These include transportation constraints, time limitations, inadequate exercise facilities [18], and environmental barriers [19].

Indian elders face various stereotypes and barriers that significantly impact their quality of life and social inclusion. The built environment plays a crucial role in shaping these experiences, particularly in terms of physical activity and mobility. One of the primary barriers faced by Indian elders is the changing family structure. The shift towards nuclear families has led to increased isolation and a lack of support for the elderly [31]. Cultural and contextual factors further constrain participation. Caste-based marginalization often limits access to safe public spaces, especially in rural regions. Rural–urban disparities are also striking: rural elders face infrastructural deficits such as poor roads and lack of exercise facilities, while urban elders often contend with sedentary lifestyles and safety concerns in crowded environments. Gender norms are equally important, as older women may face restrictions on outdoor exercise due to cultural expectations, caregiving roles, and safety issues. These socio-cultural dimensions must be addressed to ensure inclusive physical activity interventions in India. This change has resulted in economic, social, and political challenges, including physical, physiological, and emotional violence, as well as financial insecurity. The traditional reverence for elders in Indian culture is being eroded, leading to a need for protective legislation and social welfare measures [20].

The lack of indoor facilities and variable climatic conditions serve as significant barriers to physical activity [21]. These environmental factors contribute to the isolation and reduced mobility of elderly individuals. The stereotypes and barriers faced by Indian elders are multifaceted, encompassing social, economic, and environmental factors. Concurrently, the built environment plays a crucial role in either enabling or hindering their physical activity and social engagement.

## 4 Enablers & Intervention

Addressing these issues requires a comprehensive approach that includes legislative measures, social welfare programs, and improvements in the built environment to enhance the quality of life for Indian elders. The built environment significantly influences the walkability and physical activity of senior citizens in India. A study in Triplicane, Chennai, found that pedestrian safety infrastructure, physical barriers in the neighborhood, and aesthetics have a high impact on walkability among senior citizens. [22]

Online and offline physical activity programs can be gamechangers for Indian elders by addressing their unique needs and overcoming barriers to exercise participation. Physical activity programs, both online and offline, can significantly improve the health and well-being of older adults in India. These programs can help reduce the risk of falls, improve cardiovascular health, muscle strength, flexibility, and balance. They can also enhance quality of life, alleviate depression and anxiety, and boost self-esteem among Indian elders [23]. Innovative approaches like exergames, such as the Pressure-Ball game, can increase confidence in fitness levels and reduce fear of injuries, potentially leading to more sustainable and transferrable active lifestyles [24].

It is important to note that there may be challenges in implementing these programs. For instance, about one-fourth of older adults may not participate in remote exercise activities due to lack of electronic devices, internet access, or interest in remote activity formats [25].

This digital divide could be particularly pronounced in rural Indian communities. Additionally, the lack of indoor facilities and extreme climatic conditions in some areas may serve as significant barriers to physical activity [21]. To maximize the impact of physical activity programs for Indian elders, a multi-faceted approach is necessary. This could include combining online and offline options to cater to different preferences and capabilities. Home-based video exercise programs have shown positive effects on balance and strength [26], which could be particularly beneficial for those with limited mobility or access to facilities. Community-based programs, like the Southeast Senior Physical Activity Network (SESPAN), could be adapted to the Indian context to create sustainable walking and exercise programs in underserved communities [27]. Furthermore, addressing socioeconomic factors, health status, and depression, which affect exercise adherence in older adults, will be crucial for the success of these programs [28]. By tailoring these approaches to the specific needs and cultural context of Indian elders, physical activity programs can indeed be gamechangers in promoting healthy aging in India. These findings are supported by recent bibliometric evidence showing that exercise is a central strategy to reduce frailty and preserve independence among older adults [32]. For online physical activity programs, challenges include limited awareness and accessibility, difficulties in assessing performance, lack of social component, and technological issues [18]. Addressing these barriers through targeted interventions, such as community-based programs and virtual reality-based exercise [29] could potentially increase physical activity participation among older adults, leading to improved cognitive function, reduced depression risk, and enhanced social engagement.

A study published in 2016 in Lancet, the authors Guthold R et al. [30] underlines that, women were significantly less physically active than men, with 31.7% of women and 23.4% of men engaging in insufficient activity. The gender gap was most notable in Central Asia, the Middle East, North Africa, high-income Western countries, and South Asia. Promoting safe, accessible leisure activities for women could help reduce this gap.

Tailored physical activity programs for Indian elders can improve health, reduce healthcare costs, and enhance quality of life. Overcoming cultural and social barriers, such as traditional roles and lack of support, is essential for encouraging participation. The evolving research landscape in geriatric rehabilitation underlines the importance of designing evidence-based, context-sensitive policies [33]. Health policies should be culturally sensitive to drive effective behavioral change. *Integrated interventions that combine physical training with behavioral strategies have been shown to enhance both strength and mental health outcomes in older adults* ^*34*^ *reinforcing the need for a multi-pronged approach in India.*

Health policies and health promotion programs should consider these factors and be culturally sensitive to effectively promote healthy behavioral changes. These interventions could ultimately contribute to a more active, socially connected, and healthier aging population in India. Addressing environmental barriers through urban planning, inclusive public spaces, and enforcement of safety regulations is critical for sustaining elder-friendly activity programs in India. *This work aligns with the WHO Global Action Plan on Physical Activity 2018–2030, particularly in its emphasis on creating active societies, building supportive environments, empowering active people, and strengthening systems that sustain physical activity across the life course.*

## 5 Recommendations & Conclusion

Promoting active aging among Indian elders requires a holistic, culturally sensitive approach. Based on the evidence presented, the following recommendations are proposed:

1. Community-driven programs should be prioritized to reduce isolation and build social networks, which are known to enhance participation in physical activity [27].
2. Blended online and offline interventions are needed to address the digital divide. While online programs offer flexibility, community-based and home-based exercise initiatives ensure accessibility across rural and underserved populations [26].
3. Urban planning and supportive built environments must be emphasized to improve walkability, safety, and access to elder-friendly spaces ^22.^
4. Integration of caregivers and families in exercise promotion is essential, especially given the changing family structure and reduced traditional support for elders [31].
5. Policy reforms and culturally sensitive strategies, aligned with the WHO Global Action Plan on Physical Activity 2018–2030, can strengthen systems to sustain physical activity throughout later life.

## 6 Limitations & Future Research

This study focused on immediate perceptions and outcomes, without assessing long-term retention or behavior change. Future longitudinal research is needed to evaluate sustainability, adherence, and the real-world application of physical activity interventions. Moreover, further studies should examine diverse underserved populations to strengthen the generalizability and transferability of findings.

## 7 Conclusion

This study emphasizes that multiple psychological, social, cultural, and environmental barriers limit physical activity among Indian elders. Using the Social Ecological Model, it highlights the need for multi-level interventions that address family support, gender norms, rural–urban disparities, and the digital divide. Community-based initiatives, supportive urban environments, and culturally sensitive policies—aligned with the WHO Global Action Plan on Physical Activity 2018–2030—can foster active aging. Such strategies are vital to reduce frailty, enhance social connectedness, and improve quality of life in India's aging population.

## CRediT authorship contribution statement

**Nawaj Mehtab Pathan:** Writing – review & editing, Writing – original draft, Visualization, Supervision, Resources, Project administration, Methodology, Formal analysis, Data curation, Conceptualization. **Vibhuti Tiwari:** Writing – original draft, Visualization, Supervision, Software, Resources, Methodology, Formal analysis, Data curation. **Rucha Agnihotri:** Visualization, Validation, Supervision, Software, Resources, Methodology, Data curation, Conceptualization. **V. Sarath Babu:** Writing – review & editing, Validation, Supervision, Project administration, Methodology, Investigation, Conceptualization. **Prerana Dalvi:** Writing – original draft, Visualization, Validation, Supervision, Resources, Project administration, Conceptualization.

## Funding

None.

## Declaration of Competing Interest

The authors have nothing to disclose.
